# Regulation of T Cells in Cancer by Nitric Oxide

**DOI:** 10.3390/cells10102655

**Published:** 2021-10-05

**Authors:** Inesa Navasardyan, Benjamin Bonavida

**Affiliations:** Department of Microbiology, Immunology and Molecular Genetics, David Geffen School of Medicine, University of California, Los Angeles, CA 90095, USA; inesanav@g.ucla.edu

**Keywords:** T cell, nitric oxide, nitric oxide synthase, cancer, immunotherapy

## Abstract

The T cell-mediated immune response is primarily involved in the fight against infectious diseases and cancer and its underlying mechanisms are complex. The anti-tumor T cell response is regulated by various T cell subsets and other cells and tissues in the tumor microenvironment (TME). Various mechanisms are involved in the regulation of these various effector cells. One mechanism is the iNOS/.NO that has been reported to be intimately involved in the regulation and differentiation of the various cells that regulate the anti-tumor CD8 T cells. Both endogenous and exogenous .NO are implicated in this regulation. Importantly, the exposure of T cells to .NO had different effects on the immune response, depending on the .NO concentration and time of exposure. For instance, iNOS in T cells regulates activation-induced cell death and inhibits Treg induction. Effector CD8 T cells exposed to .NO result in the upregulation of death receptors and enhance their anti-tumor cytotoxic activity. .NO-Tregs suppress CD4 Th17 cells and their differentiation. Myeloid-derived suppressor cells (MDSCs) expressing iNOS inhibit T cell functions via .NO and inhibit anti-tumor CD8 T cells. Therefore, both .NO donors and .NO inhibitors are potential therapeutics tailored to specific target cells that regulate the T cell effector anti-tumor response.

## 1. Introduction

The immune system consists of both the innate and adaptive immune responses. The innate immune response consists of preexisting cells that can respond to invaders such as microorganisms, cancer, and foreign tissues as allogeneic transplants. The cells implicated in the innate response are natural killer cells (NK), macrophages, neutrophils, basophils and eosinophils [1]. The adaptive immune response consists of both the B cell humoral antibody response as well as the cell-mediated T cell response (CD4 and CD8 T cells) [1].

The T anti-tumor response mediated by the CD4 helper T cells and the CD8 cytotoxic cells are regulated by endogenous factors and other cells, such as Th17, Treg, MDSCs, etc., that will ultimately result in either a positive anti-tumor response or an immuno-suppressive response. Prior findings have implicated the role of iNOS/.NO in the regulation and differentiation of those various immune cells. For instance, Niedbala et al. [2] have reported that .NO regulated the type 17 helper T (Th17) cells that are involved in various human autoimmune diseases. .NO inhibits the proliferation and the function of the Th17 cells and mice lacking iNOS developed severe autoimmune encephalomyelitis. Lee et al. [3] have reported that .NO inhibited TGF-beta induced Th17 differentiation and the development of Treg cells. In a related study, Niedbala et al. [4] have reported that .NO induces a population of CD4^+^CD25^+^Foxp3^−^ regulatory cells (.NO-Tregs) that suppressed the functions of CD24^+^CD25^−^ effector T cells. In addition, .NO-Tregs suppressed Th17 and not Th1 cells’ differentiation and function. Xue et al. [5] reviewed the expression of iNOS in T cells, macrophages and dendritic cells and reported that .NO regulates the differentiation and function of immune cells. For example, iNOS expression in CD4 T cells inhibited the differentiation of Th17 cells. Additionally, iNOS expression by M1 macrophages is involved in the differentiation of M1 macrophages via the modulation of the signature genes in M1 macrophages. Likewise, .NO also inhibited the differentiation of effector DC cells. It is clear that the expression of iNOS/.NO in immune cells, particularly T cells, plays important roles in the differentiation and function of these immune cells, and consequently will regulate the anti-tumor immune response. Little has been reported on the role of .NO in T cells in cancer. Further, little has been reported on the roles of endogenous and exogenous .NO in the regulation of immune cells. The objective of this review was to discuss the intrinsic role of both endogenous and exogeneous .NO in the differentiation and function of immune T cells and other immune cells in the TME that regulate the anti-tumor immune response.

## 2. The Immune System

### 2.1. Overview of the Immune System

The immune system consists of both the innate and the adaptive immune responses. The innate immune response is the first line of defense against foreign invaders and spontaneous cancer. The effector cells are the natural killer cells (NK), the macrophages, the neutrophils and the eosinophils. The adaptive immune response consists of the B cell-mediated humoral antibody response and the T cell-mediated immune response. The T cell-mediated immune response is mediated by the CD4 T helper cells and the CD8 cytotoxic T cells [1,6]. The immune response is regulated by a complex set of regulatory T cells and non-T cells that are present in the tumor microenvironment (TME) [7,8]. 

### 2.2. CD4 and CD8 T Cells

The CD4^+^ T cells are often divided into T helper (Th) cells and regulatory T (Treg) cells. The Th cells represent a subpopulation of T cells responsible for eliciting an immune response by stimulating other cells, such as B cells, macrophages, and cytotoxic T cells primarily through the secretion of cytokines such as interferon gamma (IFNγ) and tumor necrosis factor-α (TNF-α) [9]. CD4^+^ T cells express T cell receptors (TCRs) that specifically recognize antigens presented by the major histocompatibility complex (MHC) class II molecules found primarily on antigen-presenting cells (APCs). The expression of MHCII on the surface of APCs has a bound antigen peptide processed by the APC cells and the complex MHCII-bound peptide is recognized by the CD4 TCR that is specific for this complex. The interaction of the TCR and the MHCII complex results in the activation of the CD4 T cells along with co-stimulatory molecules on the APCs and the corresponding receptors on the CD4 T cells [10]. Naive T cells differentiate into one of several lineages of Th cells upon T cell receptor (TCR) activation, including Th1, Th2, Th9, and Th17 cells [9]. The differentiation of naive CD4^+^ T cells into a specific lineage is dependent upon the specific network of cytokines and transcription factors involved. For instance, interleukin-12 (IL-12) and IFNγ are important cytokines involved in the differentiation of Th1 cells [11,12], whereas interleukin-6 (IL-6) and transforming growth factor beta (TGF-β) are crucial for Th17 development [13,14]. In sum, CD4^+^ T cells are crucial for their implication in the adaptive immune system and cancer immunity via their helper functions, namely their role in stimulating B cell antibody response and CD8 T cell-mediated cytotoxic response and cytokine production. Conversely, Tregs represent a subset of T cells that inhibit T cell activity and cytokine production to suppress the immune response and prevent autoimmunity to maintain self-tolerance. Recent research has found that the cytokine TGFβ is essential for Tregs to differentiate from naive CD4+ cells and is important in maintaining Treg homeostasis [15]. Treg cells were traditionally identified by high levels of CD25, whereas more recent evidence suggests identification of the transcriptional factor Foxp3^+^ as a definitive lineage regulator for Tregs [15]. The activation of Treg cells is antigen-specific, which implies that the suppressive activity of Treg cells is triggered in an antigen-specific fashion. In cancer, there has been a strong correlation between the frequencies of Treg cells in the TME and anti-tumor immunosuppression [16]. 

Cytotoxic (CD8^+^) T cells, also referred to as cytotoxic T lymphocytes (CTLs), are T cells that express the CD8 heterodimer glycoprotein on their plasma membrane. This dimeric co-receptor functions in concert with the TCR for interacting with the conserved portion of MHC class I molecules on all nucleated cells to recognize specific antigens (e.g., microbial, cancer or allogeneic transplants) [17]. Following activation by antigen-presenting cells and recognition of specific MHC-I-peptide complexes by the TCR, naive CD8^+^ T cells in conjunction with costimulatory signaling are activated and differentiate into cytotoxic effector T cells that are then able to target and destroy cells (e.g., infected cells, cancer cells) and secrete cytokines [17]. In addition, the CD8 T cells can also be activated by the Th1 cells via the secretion of IL-2 and other stimulatory molecules [18]. CTL anti-tumor activity is often referred to as being either direct or indirect. Upon interaction of CTLs with target cells, the direct mechanism by which CTLs destroy malignant cells is the result of either the production and exocytosis of cytotoxic granules (perforin and granzymes) into the target cell. This takes place when the TCR recognizes the MHC I peptide complex on the surface of the target cells and this interaction results in the microtubule organizing center (MTOC) at the junction and the formation of pores. Through these pores created by perforin, granzymes are injected into the target cells, which leads to the induction of cell death by apoptosis through the activation of the cascade pathway [19]. In addition, another mechanism of activation of the apoptotic signal is mediated by the CTL ligands (FasL, TRAIL, TNF-alpha) and the corresponding receptors on the target cells (Fas, DR4 and DR5, TNF-R) [19]. Conversely, CTLs indirectly target cancer cells via the secretion of cytokines, namely TNF-α and IFNγ, and the activation of cytotoxic macrophages and neutrophils [19]. Due to the activation of CTLs by the target cells, they become easily exhausted and inactivated and, therefore, less effective in controlling tumor progression [20]. In cancer, continuous antigen stimulation results in hyporesponsive T cell differentiation and T cell exhaustion [20]. Exhausted T cells have been shown to lead to increased expression of immune checkpoint receptors (e.g., PD-1 and other check point inhibitory receptors) and decreased cytokine production (e.g., IL-2, TNF-α and IFNγ) [21]. 

Several immunotherapeutic strategies have been developed to overcome cancer resistance to the immune response such as ex vivo activation and expansion of anti-tumor effector cells, engineered CTLs expressing tumor specific TCRs, engineering of anti-tumor CAR T cells, and administration of immune checkpoint inhibitors.

Several reports have demonstrated that, in addition to the above immune strategies, nitric oxide donors could reverse the resistance of tumor target cells to immune-mediated cytotoxic responses, both in vitro and in vivo [22]. These findings were primarily focused on how .NO sensitized tumor target cells to the cytotoxic activity of the anti-tumor CD8 T cells [23,24]. However, the role of endogenous .NO through the activation of iNOS in the T cells or the exogenous .NO through either the released .NO by T cells and other cells as well as its effect on adjacent cells and or the addition of .NO donors to the cells in the regulation of T cells have not been critically addressed. This is important from the point of view that administration of non-targeted .NO donors in vivo, in addition to targeting tumor cells, it may also affect anti-tumor T cells (CD4 and CD8), the outcome of which may or may not be highly beneficial in the eradication of the tumor.

In this review, we provide an overview of the roles of endogenous and exogenous .NO in the regulation of T cells and their subsets and their ultimate immune response.

## 3. Regulation of T Cells by .NO

### 3.1. Nitric Oxide

Nitric oxide (.NO) is a diatomic free radical molecule that occurs naturally in the human body [25]. Nitric oxide synthase (NOS) comprises a family of enzymes that can generate.NO. The three isoforms of NOS include neuronal NOS (nNOS), inducible NOS (iNOS), and endothelial NOS (eNOS) that are encoded by the *NOS1, NOS2,* and *NOS3* genes, respectively [26]. All NOS isoforms utilize the amino acid arginine and molecular oxygen as substrates as well as the co-factors NADPH, FMN, BH4, and FAD [26]. Although all three NOS isoforms have been detected in tumor samples, iNOS is the most widely studied isoform due to its significant and contrasting roles in cancer [27]. iNOS expression is regulated at the transcriptional level by cytokines (IFNγ, IL-1β, and TNF-α), bacterial endotoxins, and oxidative stress/hypoxia [28]. 

There exists both a cyclic GMP (cGMP)-dependent [29,30] as well as a cGMP-independent pathway [31,32] by which the signaling mechanisms of .NO are regulated. In the cGMP-dependent pathway, .NO reacts with the active site of soluble guanylate cyclase (sGC) to produce cGMP, which then activates Protein Kinase G (PKG) to phosphorylate downstream substrates. Conversely, the cGMP-independent pathway involves the modification of cysteine residues via S-nitrosylation, leading to either the progression or inhibition of cancer [33]. 

### 3.2. The Dual Role of .NO in the Regulation of Tumor Cell Cytotoxicity

The role of .NO in cancer is controversial, wherein .NO and NOS levels correspond with tumor suppression in some cases and tumor progression in others [34]. It was determined that the effect of .NO varies depending on its specific timing, location and concentration within the sample [34]. Additionally, the tumor microenvironment (TME) as well as the genetic background of the cancer type are also important in determining the role of .NO in either its suppression or progression. Moreover, the dual role of .NO in the mediation of tumor cell cytotoxicity is also largely based on the TME and cancer cell type [35].

The cytotoxic role of .NO has been implicated in several studies in which high levels of iNOS are associated with tumor cytotoxicity, whereas low levels may promote tumor growth and metastasis [35]. Hibbs and colleagues [36] were amongst the first to find that rodent macrophages activated by cytokines generated higher levels of .NO via increased expression of iNOS and, thus, suggested its role as an effector molecule. Li et al. [37] used murine lung vascular endothelial cells cultured and activated by cytokines IFNγ and TNF to study the role of .NO in tumor cell lysis. Endothelial cells cultured in a medium free of L-arginine, a precursor of .NO, did not result in significant tumor cell lysis, whereas those cultured with L-arginine restored nitrite concentration and tumor cell lysis [37]. This finding was further corroborated by the inhibition of L-arginine-derived nitric oxide synthesis, which led to complete inhibition of tumor cell lysis by cytokine-activated endothelial cells. Lamrani et al. [38] found that the anti-cancer drug OM-174, a toll-like receptor (TLR) 4 agonist, induces iNOS expression in murine models of breast cancer, and the inhibition of iNOS expression was found to hinder the antitumor activity of the drug, supporting the role of .NO in TLR-mediated tumor suppression. Marigo and colleagues [39] led a study demonstrating that local .NO production by tumor-infiltrating myeloid cells is important for adoptively transferred CD8(+) cytotoxic T cells to destroy tumors. Fauskanger et al. [40] studied mice bearing multiple myeloma and discovered that iNOS activity is enhanced in tumor-associated macrophages (TAMs) upon T cell recognition. The resulting distribution of .NO led to the accumulation of intracellular peroxynitrite, a much more toxic secondary oxidant formed by the reaction of .NO with superoxide radical, resulting in apoptosis of cancer cells. However, this macrophage-mediated antitumor activity by CD4+ T cells was found to be spatially restricted, wherein activated macrophages induced apoptosis of nearby cells [40]. Given the prevalence of macrophages in many solid tumors, such findings are supportive of the antitumor role of NO. The findings above, amongst others, support the role of .NO as a major mediator of cytotoxicity in tumor cells. 

Conversely, nitric oxide is also implicated in the progression of cancer. Douguet et al. [41] found that iNOS is involved in the tumor-promoting activity of γδ T cells via the inhibition of anti-tumor IFNγ and promotion of pro-tumorigenic IL-17 production, leading to metastasis. Siegert and colleagues [42] found that the human colorectal adenocarcinoma cell line HRT-18, which constitutively expresses iNOS mRNA, was more invasive than the iNOS-deficient HT-29 cell line. The induction of .NO in the HT-29 cell line via the .NO donor DETANONOate and the production of endogenous .NO led to increased invasiveness, whereas invasiveness was inhibited in both cell lines treated with an iNOS inhibitor [42]. However, it is important to consider limitations to such studies given the interaction of .NO with the relatively high concentration of oxygen in cell culture conditions. In a study led by Zhang and Xu [43], metastatic melanoma B16-BL6-bearing mice demonstrated the role of .NO in tumor progression via disrupting the host immune system to allow for tumor cell escape from immunosurveillance. Moreover, treatment of the B16-BL6 cell line with an NOS inhibitor inhibited lung metastasis, whereas treatment with L-arginine promoted metastasis of these cells [43]. Jadeski et al. [44] determined that .NO-mediated tumor growth and metastasis of the C3H/HeJ mammary tumor cell line acts via the sequential activation of NOS, guanylate cyclase (GC) and mitogen-activated protein kinase (MAPK) pathways. Ambs et al. [45] generated a human carcinoma cell line that constitutively expresses NOS2 to examine the effects of .NO production on tumor cell growth. Whereas tumor growth was inhibited in samples with wild type p53, those expressing mutant p53 experienced accelerated tumor growth, suggesting that .NO selectively promotes growth of tumors expressing mutant p53. Barreiro Arcos and colleagues [46] compared the effects of .NO on the proliferation of both normal and tumor T lymphocytes. NOS and iNOS inhibitors affected only tumor cell growth, whereas use of a calcium blocker affected only normal cell growth, indicating the role of calcium-independent iNOS expression in growing tumor cells [46]. 

These findings above demonstrated that .NO is involved in T cell mediated anti-tumor activity by two opposing effects. It was hypothesized that these contrasting effects may be due to many factors including the level of .NO, its conversion to reactive species, its stability, the cell activating status, and cytokine secretions, among other factors [47,48]. In fact, .NO produced by iNOS in macrophages and other innate immune cells is pro-inflammatory, and an essential component of the host immune response against various pathogens, including bacteria, parasites, and viruses [49]. Nonetheless, there is increasing evidence that .NO can promote immunosuppression. A significant increase in IL-12 mRNA and protein expression in iNOS KO mice (control) suggested that .NO may inhibit IL-12-mediated Th1 immune responses [50]. These findings were primarily generated in systems that did not consider the contrasting roles of .NO on T cell mediated anti-tumor response.

## 4. Expression of .NO Synthases in T Cells

**Figure 1:**
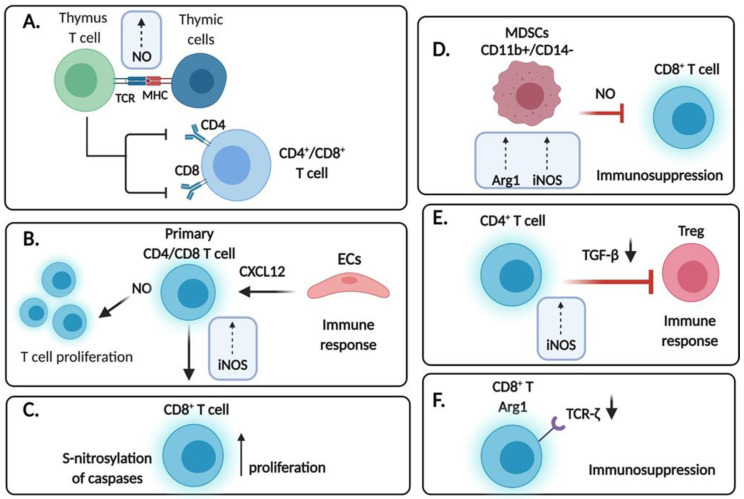
Regulation of T cells by endogenous .NO. (**A**) Increased iNOS and .NO production in thymic cells were found to induce apoptosis and depletion of double positive CD4^+^/CD8^+^ T lymphocytes. (**B**) Endothelial cells (ECs) induce iNOS expression in primary CD4/CD8 T cells, resulting in T cell proliferation likely due to CXCL12 expression and leading to an anti-tumor immune response. (**C**) iNOS expression reduces the number of T cells due to cytokine deprivation-induced cell death likely due to S-nitrosylation of caspases. (**D**) Expression of iNOS and resulting .NO in CD11b^+^/CD14^−^ MDSCs suppress the anti- tumor response of CD8+ T cells. (**E**) .NO-mediated suppression of TGF-β is due, in part, to the expression of iNOS in CD4+ T cells to inhibit differentiation of CD4 T cells into Treg cells; thus, enhancing the anti-tumor immune response. (**F**) Increased levels of the iNOS inhibitor, Arg1, results in the downregulation of the TCRζ-chain, resulting in immunosuppression.

Early studies by Tai et al. [51] investigated the role of .NO in T cell differentiation in the thymus of mice. They have found that the fetal thymus expressed the mRNA for iNOS. Performing time kinetics, they noticed that the maximal level of iNOS mRNA was at day 18 of gestation and declined thereafter after birth. In vitro analyses of a fetal thymus organ culture treated with anti-CD3 antibody resulted in the overexpression of both mRNAs for iNOS and IFNγ. The site of iNOS expression was found in the corticomedullary junction and the medulla. The treated fetal thymus organ culture with anti-CD3 induced .NO that led to the downregulation of the expressions of both CD4 and CD8 on the surface of the thymocytes. In addition, there was a significant reduction of the double positive cells and apoptosis in the cells expressing low levels of CD4 and CD8. In contrast to the double positive cells, the viability of the single positive cells was not affected and rather augmented. The authors concluded that the .NO produced following TCR stimulation in the thymus depleted the double positive cells (Figure 1A).

The role of .NO in immune function may be contradictory. The different levels of the production of .NO and the expression of iNOS may bias the balance toward immunity or immunosuppression, depending on the cell type. Jayaraman et al. [52] have reported that iNOS inhibition enhances the accumulation of Tregs in tumor-bearing mice, and a combination therapy strategy for myeloid-derived suppressor cells (MDSCs) and Tregs can be applied, which may lead to significant recovery of the body’s immune function. 

In a report by Choy et al. [53] the authors investigated the role of iNOS in the allogeneic response of T cells to endothelial cells (ECs). This study was a consequence of prior findings that indicated that iNOS expression in T cells is implicated in the pathogenesis of vascular diseases and a leading cause of mortality [54,55]. The expression of iNOS in T cells located in the arterial wall led the authors to examine whether ECs were involved in the regulation of iNOS expression in the T cells. They reported that ECs induce the expression of iNOS in primary human CD4 and CD8 T lymphocytes, though the expression was higher in the CD8 T cells. This induction was independent of TCR stimulation. The induction of iNOS was mediated by the activation of NF-kB signaling and gene transcription. The induction of iNOS and resulting .NO increased the number of proliferating T cells responding to allogeneic ECs. The EC-derived factor responsible for the induction of iNOS in T cells was not characterized though the authors ruled out serum, IL-6 and various chemokines. A follow up study by Choy et al. [56] investigated if the chemokine CXCL12 is involved in the recruitment of bystander T cells and induces iNOS expression in human CD8 T cells. The T cells may be recruited into tissues either by the stimulation of TCRs or by signaling via chemokines delivered by the vascular endothelium [57]. CXCL12, also known as stromal cell derived factor-1alpha, is involved in B cell differentiation, hematopoiesis, mobilization and recruitment of hematopoietic stem cells and upregulation of T cell responses [58,59,60,61]. The findings demonstrated that CXCL12 induced iNOS expression in human CD8 T cells and that the expression of iNOS in the infiltrated CD8 T cells is spatially associated with CXCL12 expression in human allograft arteries. Other inducers of iNOS in T cells remain to be identified (Figure 1B).

In a follow up study by Choy and Pober [62], the authors investigated the underlying mechanism by which .NO generated in bystander T cells is responsible for augmenting the accumulation of allogeneic activated CD8 T cells. They found that .NO was not responsible for the proliferation of the T cells but primarily inhibited CD8 T cell death following their activation by allogeneic ECs in vitro culture. They showed that iNOS increases the accumulation of allogeneic activated human T cells by inhibiting cytokine deprivation-induced cell death, likely through the inhibition of caspases by S-nitrosylation. Cytokine deprivation-induced cell death is a main pathway by which activated T cells are eliminated during the contraction of immune responses following pathogen infection [63,64] (Figure 1C). These findings also imply that .NO may act on cells that will affect the CD8 cytotoxic anti-tumor T cells to enhance their anti-tumor activity by inhibiting cell death via the activated induced cell death mechanism. 

Liu et al. [65] have examined both MDSCs and CD8 T cells in the peripheral blood of 173 patients with non-small cell lung cancer (NSCLC). In the naive untreated patient population, the frequency of the CD11b^+^/CD14^−^ subpopulation was increased whereas the CD8 T subpopulation was decreased. Following chemotherapy, the frequency of the MDSCs was decreased in the advanced patients who had a clinical benefit. They examined purified CD11b^+^/CD14^−^ cells from peripheral blood which expressed high levels of L-arginase 1 and iNOS. These cells suppressed the response of CD8 T cells activated with a tumor cell line. The expression and activity of iNOS/.NO in the MDSCs are in part responsible for the suppression of CD8 T cells [66]. The expression of L-arginase 1 depletes the arginine pool in the TME and results in downregulation of TCRζ expression and impaired T cell proliferation [67] (Figure 1D). Moreover, MDSCs have been shown to increase production of .NO and superoxide anions (O_2_^−^) to inhibit the activation and proliferation of T lymphocytes and induce apoptosis [68]. Moreover, .NO reacts with O_2_^−^ to produce peroxynitrite that nitrosylates the TCR and MHC. In turn, TCR-MHC I/peptide binding is disturbed and, consequently, tumor cells develop resistance to CTL-mediated apoptosis [68]. 

NOS-derived .NO is involved in the pathogenesis and control of various types of infections [47]. .NO also modulates the immune response via the regulation of apoptosis and the upregulation of cytokine expressions [69,70]. Legoretta-Herrera et al. [71] have investigated the role of .NO in CD8 T cell-induced apoptosis and cytokine production during the blood stages of malaria infection. They demonstrated, on day 11 post infection, that splenic CD8 T cells undergo apoptosis and the expression of TNF-alpha, IFNγ and IL-10 mRNAs is upregulated. Notably, the expressions of IFNγ and IL-10 in CD8 T cells was dependent on NO. Altogether, these findings indicated that .NO is involved in the regulation of CD8 T cells during the blood stages of *Plasmodium* infection.

The regulation of MDSCs induction is regulated, in part, by iNOS and iNOS is responsible for the accumulation of MDCSs in cancer and the upregulation of STAT3 and ROS that inhibit T cell activation [72]. The authors investigated the yet unknown role of iNOS expression in the regulation of Treg induction in cancer [52]. CD4 T cells expressing iNOS play a negative role in the induction of Th17 cells [73]. The authors proposed that iNOS expression in CD4 T cells limits Foxp3 expression via the suppression of TGFβ1, a regulator of the differentiation of CD4 T cells into Treg cells. The TGFβ level is significantly increased by iNOS inhibition and antibody to TGFβ abolished the induction of Treg accumulation. The molecular mechanism by which iNOS regulated TGFβ expression was not described. Overall, these findings demonstrated that iNOS expression in CD4 T cells inhibited Treg induction by inhibiting TGFβ production (Figure 1E).

Zhang et al. [74] have investigated the expression of arginase 1 (Arg1) and iNOS in the peripheral blood and lymph nodes of HIV patients. Their objective was to define the roles of Arg1 and iNOS in the progression of HIV patients. Prior studies demonstrated that the expression of Arg1 is increased [75] and the expression of iNOS is decreased [76] in the serum and PBMCs of HIV patients though the LNs findings were not reported. The findings by the authors revealed that in asymptomatic and symptomatic HIV patients the frequencies of Arg1 positive CD4 and CD8 T cells were increased, both in the peripheral blood and LNs. Interestingly, these frequencies were negatively associated with the peripheral blood CD4 T cell count and positively correlated with the HIV load. In contrast, the frequencies of iNOS positive CD4 and CD8 T cells in the periphery and the LNs were decreased. These frequencies were positively associated with the peripheral CD4 T cell count and negatively associated with the HIV load. Arg1 inhibits .NO synthesis [77] as it reduces the level of arginine leading to a reduction of .NO synthesis [78]. The decreased levels of arginine by Arg1 which causes downregulation of TCRζ-chain on CD8 T cells contributes to the immune compromising effect in HIV patients (Figure 1F).

## 5. Role of Exogenous .NO Donors on T Cells

### 5.1. Role on the Anti-Tumor T Cell Response

Roozendaal et al. [79] used the .NO-donor compounds S-nitroso-N-acetyl-D,L-penicillamine (SNAP), DPTA-nonoate (DPTA), and DETA-nonoate (DETA) to study expression of IL-2, IL-4, IL-5, and IFNγ by activated human T lymphocytes. Exogenous .NO donors added fifteen minutes prior T cell stimulation, resulting in the inhibition of IL-4, IL-5 and IFNγ, whereas IL-2 expression was not inhibited. However, a preincubation period of 24 h led to further inhibition of IFNγ and IL-2 but the initial inhibition of IL-4 and IL-5 was reversed following decay of .NO-donor compounds [79]. Using the guanylate cyclase inhibitor LY83583, Roozendaal and colleagues determined the effects of SNAP-mediated IFNγ inhibition to be cGMP-dependent, whereas the mechanisms of DPTA- and DETA-mediated inhibition of IFNγ exhibited cGMP-independent mechanisms due to their lack of response to LY83583. Decreased mRNA expression of IFNγ suggested that such mechanisms are present at the transcriptional level. Whereas IL-4 and IL-5 are associated with Th2 cells, IL-2 and IFNγ are produced by Th1 cells; it is highly probable that .NO regulates the balance between Th1 and Th2-type cytokines via IFNγ inhibition independent of cGMP.

Lee and colleagues [3] studied the role of .NO in Th differentiation of naive CD4 T cells using the two exogenous .NO donors DETA-NONOate and SNAP. Transforming growth factor beta (TGF-β) exhibits both pro- and anti-inflammatory activities and is well known for its role in the induction of Foxp3+ inducible regulatory T cells (Tregs). In the absence of .NO, TGF-β synergizes with IL-6 to induce Th17 differentiation; however, the .NO was found to antagonize IL-6 to prevent differentiation towards the Th17 lineage [3]. Exogenous .NO donors, therefore, were determined to modulate TGF-β to promote differentiation towards the Th1 lineage and antagonize Foxp3^+^ inducible Treg and Th17 differentiation, demonstrating the proinflammatory activity of TGF-β. 

### 5.2. Role of .NO in the Regulation of Checkpoint Inhibitory Receptors

**Figure 2:**
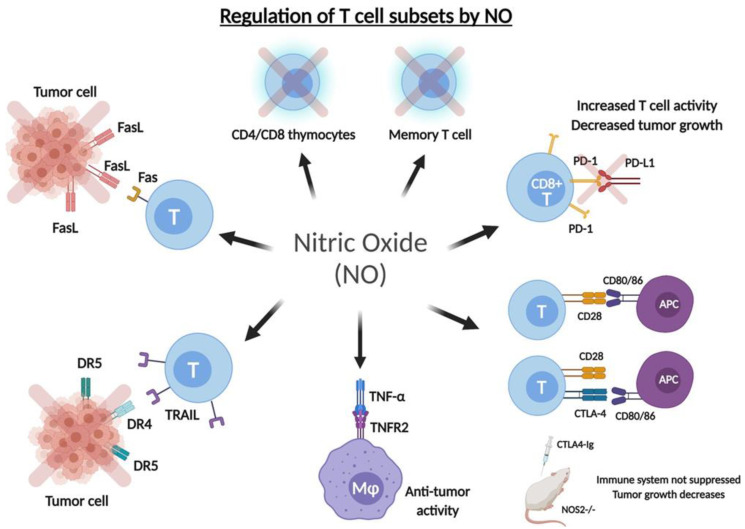
Regulation of T cell subsets by .NO. .NO is involved in the regulation of various Tcell subsets and contributes to their role in disease. Blocking PD-1/PD-L1 interactions may be due, in part, to increased levels of .NO, resulting in activation of T cell activity and subsequent inhibition of tumor growth. Interactions between CTLA4 and the CD80/CD86 ligands is found to be immunosuppressive; however, administration of the CTLA4-Ig was found to be ineffective for immunosuppression in .NOS2-deficient mice, suggesting a role of .NO in CTLA4-mediated suppression of T cell activity. .NO production is necessary for the interaction between TNF-α and TNFR2 in suppressing tumor growth. .NO production sensitizes tumor cells to FasL-mediated apoptosis, whereas inhibition of .NO evades FasL-mediated apoptosis and promotes tumor progression. Increased .NO production leads to upregulation of the TRAIL ligand as well as its receptors to sensitize tumor cells to TRAIL-mediated apoptosis. .NO is found to reduce expression of CD4^+^/CD8^+^ thymocytes as well as memory T cells.

Programmed cell death protein 1 (PD-1) is found on the surface of activated T cells and has been shown to be upregulated in the anti-tumor CD8 T cells in many cancers. The interaction between PD-1 and its ligand (PD-L1) prevents T cells from destroying cancer cells and, thus, promotes tumor progression. The mechanism by which PD-1/PD-L1 acts to inhibit the cytotoxic activity of CD8 T cells is via the inhibitory cell signaling triggered by PD-L1 on the tumor and the inhibitory PD-1 receptor on the CD8 T cells. This inactivation results in decreasing cell proliferation, cytokine activity, T cell survival and T cell exhaustion [80]. Monoclonal antibodies targeting either PD-1 (nivolumab, pembrolizumab) or PD-L1 (atezolizumab, avelumab, durvalumab) effectively remove these brakes on the immune system to promote an effective immune response in cancer [81]. PD-1 is expressed both on exhausted as well as effector CD8 T cells [82,83]. Upon T cell activation, PD-1 levels were found to be rapidly increased, and the inhibition of PD-1 resulted in improved CD8 T cell effector function during viral infection [83]. One study in NSCLC patients with and without COPD found increased levels of exhaled .NO in patients treated with the PD-1 inhibitor nivolumab [84]. Furthermore, the transcription factor Yin-Yang 1 (YY1) is known to further enhance the effects of PD-L1; therefore, .NO-mediated inhibition of YY1 and subsequent downregulation of PD-L1 further enhances the immune response in cancer [23]. Given the above findings, one mechanism by which PD-1 inhibition may enhance CD8 T cell effector function may be due to increased levels of .NO production (Figure 2).

The CTLA-4 (CD152) co-receptor is an extremely potent inhibitor of T cell function that acts by strongly binding co-stimulatory molecules on antigen-presenting cells to prevent binding of these molecules and subsequently inhibiting T cell activation. Local .NO production by tumor-infiltrating myeloid cells plays a significant role in adoptively transferred CD8(+) cytotoxic T cells in destroying tumors [39]. CTLA-4 interacts with CD80 (B7–1) and CD86 (B7–2) co-stimulatory molecules which are necessary for binding CD28 co-stimulatory receptor to induce priming of the immune response. Although CD28 is more highly expressed, CTLA-4 has a higher binding affinity than CD28 for the CD80 and CD86 ligands. Ipilimumab was the first approved anti-CTLA-4 blocking antibody in the treatment of cancer patients. Although constitutively expressed by regulatory T cells (Tregs), CTLA-4 also plays a role in CD8 T cell function. One study examining the effects of CTLA-4 blockade on memory CD8 T cells found that inhibition of CTLA-4 led to increased expansion and enhanced CD8 T cell effector function, including increased production of effector cytokines IFNγ and TNF-α [85]. The immunosuppressive drug CTLA4-Ig prevents binding of CD28 to either CD80 or CD86 and, thus, disrupts the priming stage by inhibiting costimulatory signals to T cells. However, one group found that CTLA4-Ig was effective in suppressing the immune response in CD28-deficient mice but ineffective in NOS2-deficient mice; thus, CTLA4-Ig was determined to inhibit the immune response independently of CD28 via a NOS2-dependent mechanism [86]. Although the specific mechanism by which CTLA4-Ig regulates NOS activity remains largely unknown, NOS levels may prove to be crucial in effective T cell function (Figure 2).

### 5.3. Role of .NO in the Expression of TNF-Alpha, Fas and TRAIL on T Cells

#### 5.3.1. Tumor Necrosis Factor-α (TNF-α)

Tumor necrosis factor (TNF) acts as both a pro-inflammatory and anti-inflammatory cytokine in many diseases and its effects are mediated by the TNF receptor type I (TNFR1) and TNF receptor type II (TNFR2) receptors, with TNFR2 being highly implicated in maintaining a balance between the regulatory and the effector T cells [87]. Zhao et al. [88] showed that TNF activated TNFR1^−/−^ macrophages to produce .NO, likely via TNFR2, to elicit its antitumor activity. Moreover, treatment of TNFR1 knockout mice with the L-NAME NOS inhibitor resulted in elimination of TNF-induced tumor suppression, suggesting that .NO production is necessary for TNFR2-mediated suppression of tumor growth likely due to the mediation of regulatory and effector T cells [88] (Figure 2). Furthermore, it has been shown earlier that treatment of cells with NO inhibits NF-κB activity [89]. Provided that TNF-α is a target of NF-κB, therefore, NO-mediated inhibition of NF-κB will result in the inhibition of TNF-α expression in T cells [90].

#### 5.3.2. Fas/FasL

Garban and Bonavida [91] explored the role of .NO in the sensitization of cancer cells to Fas-mediated apoptosis via IFNγ expression as well as the role of .NO in the expression of the Fas receptor (Fas). Given that IFNγ stimulation resulted in the induction of cell surface Fas expression as well as the induction of iNOS and .NO, it was hypothesized that FasL-mediated apoptosis may be due, at least in part, to .NO. Supportively, the .NO donor SNAP sensitized tumor cells to FasL, while the .NO inhibitor L-NAME evaded FasL-mediated apoptosis by IFNγ [91]. The Yin Yang 1 (YY1) transcription factor is known to negatively regulate the expression of the Fas receptor and, thus, is implicated in tumor cell resistance to Fas-induced apoptosis [92]. Moreover, treatment with .NO donors revealed that .NO inhibits YY1 expression and DNA-binding activity via inhibiting the upstream NF-κB pathway as well as through S-nitrosylation of YY1 [93]. Thus, .NO may mediate FasL-induced apoptosis via inhibition of the Fas repressor YY1 (Figure 2).

#### 5.3.3. TRAIL

Tumor necrosis factor-related apoptosis-inducing ligand (TRAIL), a member of the TNF family of ligands, selectively triggers cancer cell apoptosis via interaction with the DR4 and DR5 death receptors [94]. Primary human endothelial cells cultured with TRAIL resulted in enhanced phosphorylation of eNOS as well as increased NOS activity and, in turn, increased .NO synthesis [95]. Huerta-Yepez et al. [96] treated prostate cancer cell lines with the .NO donor DETANONOate to sensitize cells to TRAIL-induced apoptosis via inhibition of the oncogenic NF-κB pathway and subsequent downregulation of the Bcl-2 related gene *(Bcl-_xL_)*. Moreover, Huerta-Yepez and colleagues [97] found that DETANONOate sensitized TRAIL-resistant tumor cells to apoptosis via upregulation of DR5. YY1 was suggested to be a negative regulator of DR5 expression, which was supported by the finding that cells treated with YY1 siRNA resulted in increased DR5 expression and, in turn, increased TRAIL-induced apoptosis [97]. In contrast, the interactions between TRAIL and its receptor have resulted in the direct suppression of T cell activation via inhibition of TCR signaling [98] and, therefore, the role of .NO in TRAIL expression may suggest the possible role of .NO in TRAIL-mediated immunosuppression (Figure 2).

## 6. Role of .NO in the Activation of Induced Cell Death of T Cells

Previous investigations of mouse thymocytes treated with .NO donors revealed the pro-apoptotic role of .NO in the depletion of CD4^+^CD8^+^ thymocytes [99]. Moreover, Williams and colleagues [100] demonstrated the role of NOS in the apoptotic cell death of mature T cells following TCR stimulation. They also showed that inhibition of NOS protected T cells from cell death in a FasL-dependent manner. 

Saio and colleagues [101] studied the role of .NO production on the induced cell death of T cells by tumor associated macrophages (TAMS). TAMs express cell surface-associated type I (CD120a) and type II (CD120b) TNF receptors to induce the release of IFNγ and the subsequent secretion of .NO upon interaction with T cells, resulting in T cell apoptosis. Supportively, TAMs isolated from mice deficient in either type I or II TNF receptor did not produce .NO nor induced T cell apoptosis following interaction with activated T cells [101]. Evidence supported the role of both TNF receptors in .NO production, suggesting that both receptors must be expressed on macrophages to elicit an apoptotic response. Furthermore, inhibition of iNOS in T cell-TAM cocultures prevented induced cell death of T cells. 

Vig et al. [102] found that iNOS-deficient mice yielded increased numbers of CD4 and CD8 memory T cells following immunization, suggesting the role of .NO in T cell death and immune memory. Moreover, iNOS^−/−^ mice exhibited reduced post-activation T cell death, increased expression of anti-apoptotic proteins Bcl-2 and Bcl-xL, and higher frequency of cytokine-secreting CD4^+^ and CD8^+^ T cells. Supportively, the use of inhibitors targeting iNOS resulted in reduced T cell death following immunization in both mouse and human T cells, leading to enhanced memory responses [102] (Figure 2).

## 7. Role of .NO in the Regulation of T Regulatory Cells (Tregs) and Dendritic Cells (DCs) in the TME

Solid tumors are present in an environment called the tumor microenvironment (TME). The TME consists of a heterogeneous population of cells including the stromal cells, and the extracellular matrix components (non-cellular), cancer cells, immune lymphoid cells (CD8 T cells, CD4 T cells, Tregs, NK cells, B cells), macrophages (M1) and tumor associated macrophages (M2), myeloid derived suppressor cells (MDCS), neutrophils, cancer-associated fibroblast cells and endothelial cells. These various components interact with each other via cell-to-cell contacts and via secreted factors. In addition, they provide the nutrients for cancer survival and growth and regulate the tumor cell resistance to cytotoxic therapies. The TME is usually an immunosuppressive milieu, and the anti-tumor cytotoxic cells are usually inactive and exhausted [8,103,104]. 

**Figure 3:**
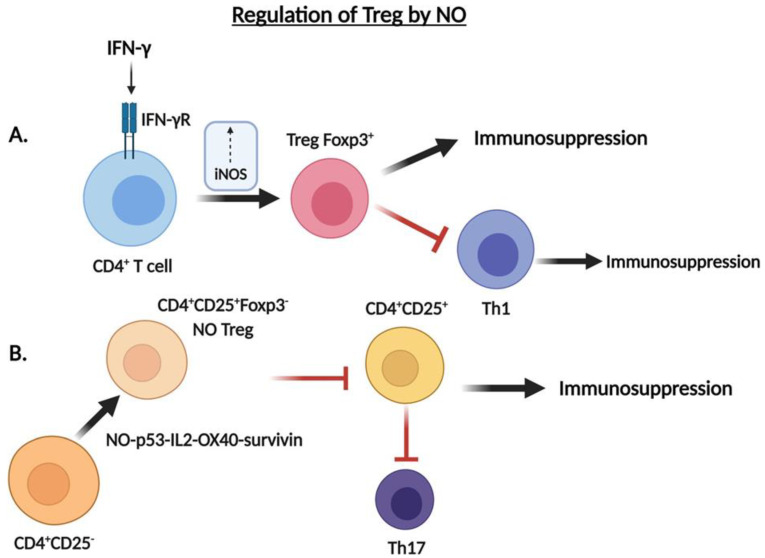
Regulation of Tregs by .NO. (**A**) IFN-γ induces a population of Foxp3+ Tregs in an iNOS-dependent pathway to exert its immunosuppressive role in the TME. (**B**) The emergence of CD4^+^CD25^+^Foxp3^−^ .NO Tregs is a result of the conversion of CD4^+^CD25^−^ T cells via a .NO- p53-IL2-OX40-survivin signaling pathway. .NO-Tregs serve an immunoregulatory role via suppression of .NO-mediated activation of Th17 cells during infection.

Although traditionally thought to act as a pro-inflammatory cytokine, IFNγ also demonstrates an immunoregulatory role in the immune system. CD4^+^ T cells conditioned with IFNγ exhibited increased numbers of Foxp3^+^ Tregs via the conversion of non-Treg precursors into functional Foxp3^+^ T regs [105]. Introduction of a NOS inhibitor, N-methyl-L-arginine (L-NMMA), resulted in the inhibition of the Treg response, suggesting the role of .NO in the immunosuppressive activity of IFNγ. Real time PCR analysis revealed a positive correlation between iNOS and Foxp3^+^ expression, contrary to findings reported by Jayaraman et al. [52]. Introduction of the .NO donor SNAP restored the IFNγ effect even in the absence of IFNγ, further supporting the role of .NO in IFNγ-mediated immunosuppression [105] (Figure 3A). These findings suggest that .NO-mediated regulation of the Tregs immunosuppressive function results in the inhibition of the CD8 T anti-tumor effector cytotoxic cells, leading to tumor progression. 

In a previous study, Niedbala et al. [106] reported that the induction of CD4^+^CD25^+^Foxp3^−^ from CD4^+^CD25^−^ T cells is regulated via the .NO-p53-IL-2-OX40-survivin signaling pathway. .NO-Tregs represent a particular population of CD4^+^CD25^+^Foxp3^−^ regulatory T cells, which have shown to exert immunosuppressive effects via suppression of CD4^+^CD25^−^ effector T cell function [4]. .NO-Tregs were found to selectively inhibit Th17 but not Th1 cell differentiation and activity, whereas the opposite held true for natural Tregs [4]. Th17 cells are highly implicated in the pathogenesis of various autoimmune diseases and cancer via production of inflammatory cytokines, such as IL-17; however, Th17 cells also play a role in the anti-tumor response via the recruitment of immune cells into tumors, stimulation of effector CD8^+^ T cells, and transformation into the Th1 phenotype [107]. Th1 cells, on the other hand, are largely characterized by their production of IFNγ, IL-2 and TNF-α to induce cell-mediated immunity and inflammation [108]. .NO-mediated activation of Th17 cells during infection may lead to a hyperactive immune response leading to a range of autoimmune disorders; thus, the role of .NO-Tregs in attenuating the immune response is consistent with its selective inhibition of Th17 [4] (Figure 3B).

**Figure 4:**
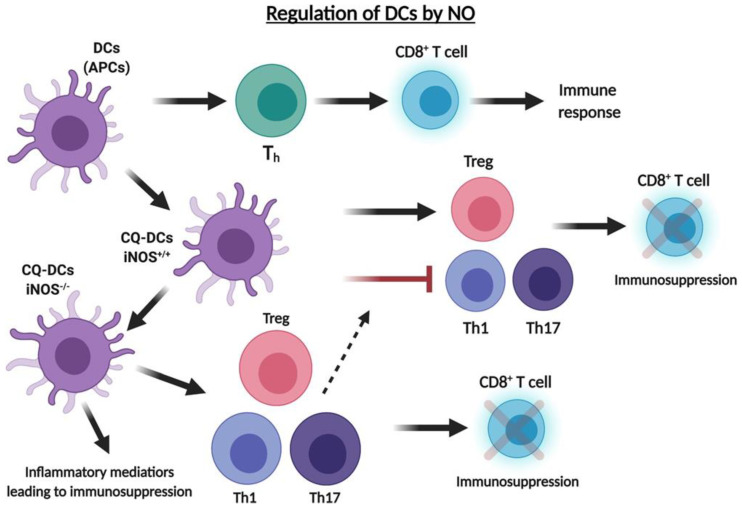
Regulation of DCs by .NO. Dendritic cells (DCs) act as antigen presenting cells to activate T helper cells to induce an immune response. Wild type chloroquine DCs (CQ-DCs) with iNOS +/+ promotes the activation of Tregs, which leads to CD8 T cell immunosuppression. Additionally, CQ-DCs simultaneously inhibit the differentiation of Th1/Th17 cells leading to immunosuppression. CQ-DCs with knockout iNOS promotes the differentiation of Treg, Th1 and Th17 cells leading to immunosuppression. They also result in the induction of inflammatory modulators to promote inflammatory tumor progression.

Dendritic cells (DCs) act as antigen-presenting cells (APCs) that mediate the host immune response via activation of naive T cells. Tolerogenic dendritic cells are a particular form of DCs used in the treatment of inflammatory diseases. Verinaud et al. [109] used chloroquine (CQ)-DCs to acquire a tolerogenic phenotype in the study of experimental autoimmune encephalomyelitis (EAE), a T cell-mediated autoimmune disorder regulated by Th1/Th17 cells. Wildtype CQ-DCs promoted the differentiation of Tregs and suppression of Th1/Th17 cells. Conversely, iNOS^−/−^ DCs did not exhibit the tolerogenic phenotype, rather it promoted the differentiation of Tregs as well as Th1/Th17 cells [109]. iNOS^−/−^ CQ-DCs resulted in increased expression of inflammatory modulators and, thus, were ineffective in suppressing the development of EAE. Thus, it was concluded that .NO played a pivotal role in mediating the function of tolerogenic DCs in the treatment of inflammatory diseases (Figure 4).

## 8. Role of .NO in the Regulation of Myeloid Derived Suppressor Cells (MDSCs) in the TME

**Figure 5:**
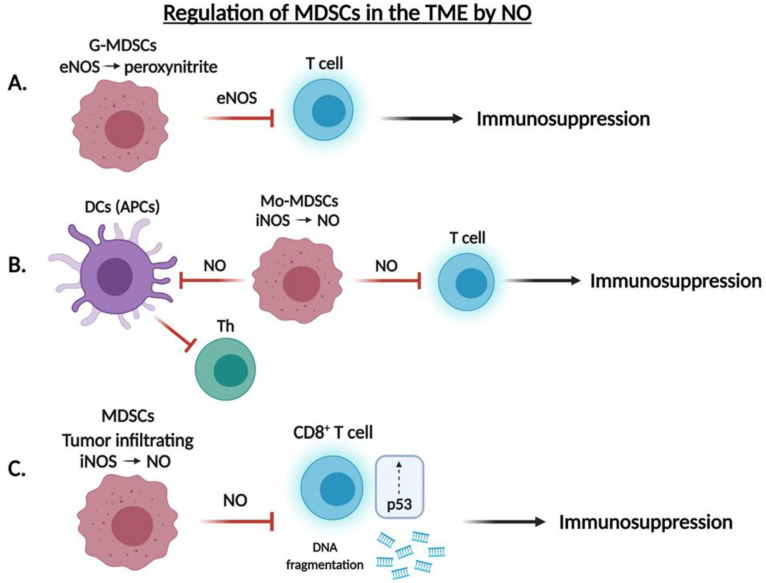
Regulation of MDSCs in the TME by .NO. (**A**,**B**) Granulocytic MDSC (G-MDSC) and monocytic MDSC (Mo-MDSC) inhibit T cell function via the eNOS-mediated generation of peroxynitrite and iNOS-mediated generation of .NO, respectively. (**C**) Tumor infiltrating MDSCs inhibit T cell function via DNA fragmentation and induction of the p53 pathway in CD8^+^ T cells via an iNOS-dependent pathway.

The largest group of nucleated cells of hematopoietic origin consists of myeloid cells, which are an important component of the innate immune system. In cancer, however, the myeloid cells are reprogrammed by tumor-derived factors to suppress the anti-tumor immune response to promote tumor growth. The presence of myeloid derived suppressor cells (MDSCs) in tumor-bearing samples is associated with tumor progression in cancer, including their crucial role in inhibiting the T cell response. Raber et al. [110] found that granulocytic MDSCs (G-MDSC) inhibit T cell function depending on eNOS-mediated generation of peroxynitrite, whereas monocytic (Mo-MDSC) subgroups of MDSCs blocked T cell function via the iNOS-mediated generation of .NO. Many additional mechanisms have been reported on the immunosuppressive functions of MDSCs. For instance, MDSCs can interact with the T cells through their inhibitory receptors and inhibit their function. In addition, MDSCs express high levels of Fas ligand that can induce apoptosis of the infiltrating CD8 T cells expressing Fas. Additionally, MDSCs upregulate check point inhibitors (e.g., PD-1, CTLA-4, TIM3) on T cells. MDSCs express high levels of indoleamine 2,3-dioxygenase 1 (IDO1) that deplete L-tryptophan and kynurenine production and lead to T cell death. MDSCs also generate nitrogen reactive species (RNS) peroxynitrite through the reaction of .NO and O2—inhibiting the recruitment of tumor specific T cells and leading to tumor progression [111]. Targeting of either the G-MDSC or the Mo-MDSC mechanism did not affect T cell suppression by the other pathway, suggesting that these two mechanisms acted independently to regulate T cell activity (Figure 5A,B).

MDSCs expand during various pathological diseases to produce reactive oxygen and nitrogen species involved in the mediation of T cell responses [112]. Dendritic cells (DCs) are known for their role in presenting cancer cell antigens to CD4^+^ T cells via the JAK/STAT signaling pathway [113]. MDSCs isolated from the peripheral blood of cancer patients were found to express increased levels of .NO, suggesting that MDSCs may inhibit the dendritic cell immune response via an .NO-mediated mechanism [112]. Two .NO donors (SNAP and DETA-NONOate) were used to inhibit antigen presentation by DCs; conversely, two iNOS inhibitors (L-NAME and NCX-4016) restored T cell proliferation [114].

More recently, Cartwright et al. [115] found that tumor infiltrating MDSCs induce DNA damage and p53 activation in CD8^+^ T cells in an iNOS-dependent pathway. In the presence of small molecule inhibitors of iNOS or inactivation of the *Nos2* gene in MDSCs, DNA damage of T cells was reduced and, thus, suggested the specific role of the iNOS isoform in inducing DNA damage of CD8^+^ T cells. Thus, whereas the early stages of T cell activation remain unaffected in the presence of MDSCs, induction of DNA damage and p53 pathway activation via iNOS may account for the inhibition of T cell function and, in turn, decreased survival outcomes in cancer patients (Figure 5C).

## 9. Future Perspectives and Conclusions

Chimeric antigen receptor T cells (CAR-T cells) are genetically modified T cells produced with artificial T cell receptors used to target specific cancer cell antigens [116]. Tumor associated macrophages (TAMs) are tumor infiltrating inflammatory cells that promote tumor progression and evasion of the immune system largely due to the suppression of T cells and CAR-T cells. One mechanism by which TAMs promote cancer cell resistance to chemotherapeutic agents, such as cisplatin, is via the production of .NO generated by iNOS of M2-polarized TAMs [117]. 

The efficacy of CAR-T cells is limited by the resulting toxicity of the cytokine release syndrome (CRS). Proinflammatory M1 macrophages play a role in the pathogenesis of CRS and such macrophages were found to express significant amounts of iNOS following CAR-T cell treatment in vivo. Moreover, administration of an iNOS inhibitor in cases of severe CRS helped reduce toxicity and improve survival [118]. IL-6 and IL-1 are two inflammatory cytokines largely involved in CRS. Both IL-6 and IL-1 are known to induce iNOS expression and inhibition of both IL-6 and IL-1 alleviates CRS via reduction of the proportion of iNOS+ macrophages and, thus, provides further support for the role of iNOS in the pathogenesis of CRS following CAR-T cell treatment [119,120].

The role of iNOS/.NO in the regulation of T cell subsets and their functional activities is complex due to the pleiotropic activities of .NO as well as the conditions in which it is involved. Clearly, the concentration of .NO, the stability, the triggering signals for its synthesis, the cells involved and its source, its intracellular and extracellular effects, etc., play major roles on the outcome manifested by the various T cell subsets. Several reports in the literature have discussed the roles of iNOS and .NO in T cell differentiation in the thymus and the periphery, its role in the regulation of CD4 and CD8 T cells, Th1, Th2, and Th17 and immunity to infections, cancer and autoimmune diseases [5,121,122,123,124]. 

iNOS function not only contributes to pathogen killing, but also has immune-regulatory effects, such as inhibiting or activating T cell activities. However, it is unclear whether all immune T cell subsets express iNOS under non activating conditions and how iNOS expression regulates T cell differentiation and function. Additionally, it is not clear how iNOS-derived .NO regulates various transcription factors by immune cells and how iNOS-.NO regulates the epigenetic mechanisms in gene transcriptions required for T cell functions. Future studies are needed to investigate these important and unresolved issues.

**Figure 6:**
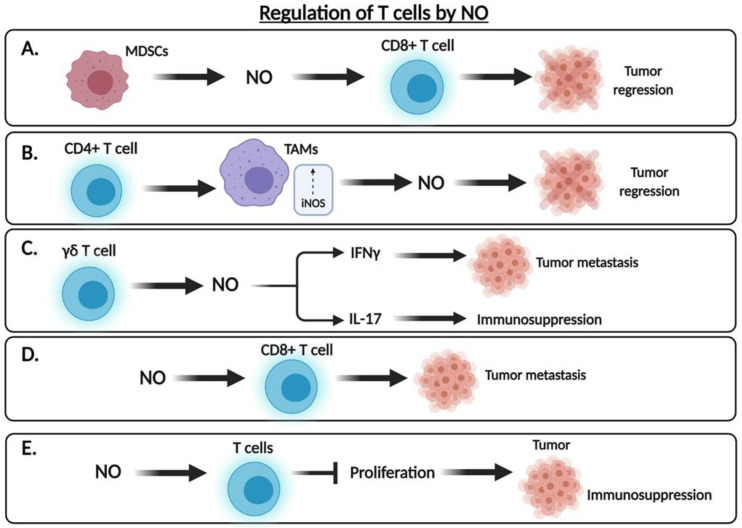
Regulation of T cells by .NO. Various subsets of T cells and immune cells are regulated by .NO. (**A**) The myeloid-derived suppressor cells (MDSCs) release .NO that can act on anti-tumor CD8T cells to activate their functions, resulting in tumor regression. (**B**) CD4 T cells interact with TAMS in the TME and activate iNOS, resulting in the release of .NO that may directly target and kill tumor cells. (**C**) The high level of iNOS in the gamma-delta T cells release .NO that will act on the inhibition of IFN-gamma that leads to tumor metastasis. In addition, .NO will activate Th17 cells that will result in immunosuppression. (**D**) Available .NO in the TME will act on anti-tumor CD8T cells and inhibit their functions and lead to tumor progression and metastasis. (**E**) Available .NO will act on T cells and inhibit their proliferation leading to immunosuppression.

.NO can have contrasting effects as either an immunoactivator or an immunosuppressive mediator. The underlying mechanisms for these contrasting activities are complex and not fully understood. In depth studies that deal with the metabolic and biochemical mechanisms are needed and should provide clear guidelines of the underlying causes responsible for .NO-mediated immunoactivation or immunosuppression. Such studies are relevant for the development and translational application of .NO donors/inhibitors as therapies for immune-mediated diseases and cancer (Figure 6).

The contrasting roles of .NO in tumor immunity is the result of its interaction between its concentration and the time span in the tumor microenvironment as well as its biochemistry, physiology and pathology as a function of tumor progression. Importantly, given the dual effects of .NO in tumor immunology, it is primordial first to determine the underlying mechanisms of the regulatory functions of .NO before its therapeutic application using it alone or in combination with current immunotherapeutic and targeted therapeutics. Furthermore, the potential use of .NO donors or inhibitors in modulating the T cell mediated anti-tumor response may be problematic since .NO donors or inhibitors will not be specifically targeting the desired T cell but will also act on the tumor cells and the TME and the outcome will be unpredictable. Targeting directly iNOS in the desired T cells will require the engineering of complexes with anti-T cell specific antibody or other nanotechnologies. Future preclinical studies need to be undertaken to test these therapeutic strategies.

## Data Availability

Not applicable.

## Abbreviations

APC	Antigen-presenting cell
Arg1	Arginase 1
CAR-T	Chimeric antigen receptor *T* cell
CTL	Cytotoxic T lymphocyte
DC	Dendritic cell
EC	Endothelial cell
eNOS	Endothelial NOS
IFN-γ	Interferon-γ
iNOS	Inducible NOS
MDSC	Myeloid derived suppressor cell
nNOS	Neuronal NOS
NO	Nitric oxide
NOS	Nitric oxide synthase
PD-1	Programmed cell death protein-1
TAM	Tumor-associated macrophage
TCR	T cell receptor
TGF-β	Transforming growth factor beta
Th	T helper
TME	Tumor microenvironment
TNF-α	Tumor necrosis factor-α
TNFR1	TNF receptor 1
TNFR2	TNF receptor 2
TRAIL	Tumor necrosis factor-related apoptosis-inducing ligand
Treg	T regulatory
YY1	Yin-yang 1
